# Growth of *Hydrogenophaga pseudoflava* on syngas: design of gas and liquid medium composition based on elemental yield coefficients

**DOI:** 10.3389/fbioe.2025.1727931

**Published:** 2026-01-07

**Authors:** Florian Miserez, Sven Panke, Manfred Zinn

**Affiliations:** 1 Institute of Life Sciences, University of Applied Sciences and Arts Western Switzerland (HES-SO Valais-Wallis), Sion, Switzerland; 2 Department of Biosystems Science and Engineering, ETH Zurich, Basel, Switzerland

**Keywords:** aerobic cultivation, design of experiment, gas and medium optimization, *Hydrogenophaga pseudoflava*, syngas

## Abstract

Syngas, an industrial byproduct composed of H_2_, CO, and CO_2_, represents an energy-rich substrate for sustainable bioprocesses. However, the toxicity of CO limits its biological utilization to a small number of microorganisms, primarily cultured under anaerobic conditions. To expand the applicability of syngas in aerobic systems, this study investigates the use of *Hydrogenophaga pseudoflava* as a CO-oxidizing strain capable of converting CO into CO_2_. This strain has been reported to be a suitable host strain for the engineered biosynthesis of value-added compounds such as the C_15_ sesquiterpene. The amount of product synthesized depends directly on the amount of CO converted to CO_2_ and subsequently assimilated as a carbon source. Under previously reported culture conditions, only low CO_2_ production rates were achieved. To enhance CO oxidation and CO_2_ generation as a performance indicator, both the gas feed composition and the culture medium were optimized, leading to a 75% increase in specific growth rate (0.072 h^-1^) and a 380% increase in biomass concentration (11 g L^−1^). These improvements resulted in a twofold increase in volumetric CO_2_ production rate. Altogether, our findings establish *H. pseudoflava* as a promising platform organism for sustainable syngas bioprocesses and provide a base for further metabolic and process engineering toward bioplastic or biofuel production being part of a circular economy.

## 1 Introduction

Syngas (also known as synthesis gas) is a mixture typically composed of 25%–30% H_2_, 30%–60% CO, and 5%–15% CO_2_ (Achinas et al., 2022) making it a carbon-rich gas that holds significant potential for both chemical and microbial processes. Its worldwide global production reached 598 million of tons in 2018 (Schreiber et al., 2020). Syngas is mainly produced from coal (48%) and natural gas (47%) as an intermediate product for chemical synthesis. The primary sources of syngas as a waste are steel milling and petroleum refining, which together account for approximately 4.3% of the total syngas production (Schreiber et al., 2020), making syngas an attractive target for the valorization of waste products and the defossilization of industry including employment of renewable resources for its production through gasification (representing less than 1% of the syngas production) (Detz et al., 2024). In industrial chemistry, it is used to synthesize methane (Rönsch et al., 2016), acetic acid (Smith, 1986), and many different molecules for transportation and energy storage using variants of the Fischer Tropsch process (Keunecke et al., 2024). However, these processes are energy intensive due to the required high reaction temperature (between 600 °C and 800 °C) (Fu et al., 2019) and elevated pressure (more than 200 bar) (Mohsenzadeh et al., 2017).

Syngas is also employed as a feedstock in biotechnological processes aimed at reducing greenhouse gas emissions (Handler et al., 2016) by cultivating microorganisms under anaerobic and chemoautotrophic conditions (Neto et al., 2025). Typically, the H_2_ serves as a source of reduction equivalents, whereas CO is used as carbon source for the biomass production, for example, by reduction through the Wood–Ljungdahl pathway to produce the central intermediate acetyl-CoA (Bengelsdorf et al., 2013).

Importantly, a syngas-based bioprocess can be a more sustainable alternative to its chemical counterpart (Handler et al., 2016). For example, syngas-based bacterial production of acetate, ethanol, or butyrate takes place under much milder conditions and yields less toxic waste (Gavrilescu and Chisti, 2005).

One important challenge for a more extended use of syngas biotechnologically is the toxicity of CO for respiring organisms (including humans) (Hanley and Patel, 2022; Sigel et al., 2014). In fact, even in CO-tolerant bacteria, CO can significantly inhibit biomass and product formation. For example, in a batch culture of *Cupriavidus necator*, biomass yield decreased six-fold when the CO concentration in a gas mixture (including H_2_, CO_2_, O_2_, and N_2_) was raised from 0% to 10% (Tanaka et al., 2011). Syngas is also flammable with O_2_ due to the presence of CO and H_2_ (CO has a lower flammable limit at 12% (v v^−1^) and H_2_ of 6% (v v^−1^) in air at 20 °C and at atmospheric pressure) (Keçebaş and Kayfeci, 2019; Cohen, 1992).

In line with the inhibition of respiration mentioned above, most syngas-consuming bacteria grow under anaerobic conditions, including acetogenic and hydrogenogenic bacteria. Acetogens are well-studied and already employed in industry for ethanol production, such as the LanzaTech process (Medeiros et al., 2020). These organisms, including *Clostridium autoethanogenum*, *C. ljungdahlii,* and *C. ragsdalei* (Köpke et al., 2011), as well as thermophilic species like *Carboxydothermus hydrogenoformans* (Dobbek et al., 2001), and *Moorella thermoacetica* (Darnault et al., 2003). Some *Clostridium* species are capable of producing different chemical compounds like acetone (Kato et al., 2021). *Rhodospirillum rubrum* is a hydrogenogenic bacterium that can convert syngas directly into the biopolymer polyhydroxybutyrate (PHB) (Drennan et al., 2001; Rodríguez et al., 2021)), though only at low content (ca. 0.3 g(PHB) g(DCW)^−1^) (Karmann et al., 2019).

However, anaerobic industrial bioprocesses are also challenging as already the presence of trace amounts of O_2_ may have a negative impact on the CO dehydrogenase enzyme (CODH) of the Wood-Ljungdahl pathway. This enzyme catalyzes the reduction of CO_2_ into CO and its reverse reaction, ensuring the supply of CO_2_ that feeds into the complex of CODH and acetyl-CoA synthase that assimilates *in situ*-formed CO into acetyl-CoA (Can et al., 2014), which in turn is essential for biomass production. The CODH in this complex is composed of a cluster containing Ni and Fe, which have a stronger affinity to O_2_ than CO, so that growth is impaired in the presence of oxygen (Dobbek et al., 2001). As a result, a number of potential waste gas streams is excluded from acting as a CO-source (Siebert et al., 2022).

Carboxydotrophic bacteria that can use CO under aerobic conditions therefore follow a different metabolic strategy: They also oxidize CO to CO_2_ using a CODH, but this is an enzyme that uses a copper-molybdenum-pyranopterin complex, which is less sensitive to oxygen (Can et al., 2014). Furthermore, CODH does not play a role in carbon assimilation, which instead proceeds through RuBisCo and the Calvin-Benson pathway. Also, their respiratory chain is more tolerant to CO, because their specific cytochromes have a higher affinity to O_2_ than to CO (Cypionka et al., 1985). Finally, these bacteria can generate indirectly reduction equivalents from the oxidation of CO to CO_2_ and from the oxidation of H_2_ via a soluble hydrogenase (Siebert et al., 2022).

One such carboxydotrophic bacterium is *Hydrogenophaga pseudoflava* DSM 1084 (formerly *Pseudomonas pseudoflava* and *Pseudomonas carboxydoflava*) (Willems et al., 1989), a yellow pigmented Gram-negative bacterium isolated from water of the Moskva River in 1977 (Zavarzin and Nozhevnikova, 1977). It is a facultatively autotrophic and obligately aerobic bacterium with a fully sequenced genome (Pavan et al., 2022). A genetic engineering toolbox was developed for the production of (E)-α-bisabolene, a starting material for the synthesis of many natural products (Grenz et al., 2019). Although *H. pseudoflava* has considerable biotechnological potential, its physiology, particularly under CO-rich growth conditions remains limited, suggesting that relevant cultivation parameters could yet be substantially optimized, potentially enabling more efficient syngas-based production processes under aerobic conditions, even while respecting the limitations imposed by safety considerations with regard to flammability (Börner, 2016; Kirchman, 2011).

In this study, we enhanced the maximum specific growth rate of *H. pseudoflava* and improved its CO uptake and CO_2_ production. To achieve this, we systematically investigated the effects of gas feed and liquid medium compositions on these parameters and developed a model describing the relationship between the specific growth rate and both gas and medium compositions. This work is the first to quantitatively analyze elemental biomass yield coefficients and to elucidate the combined effects of gas and liquid compositions on aerobic carboxydotrophic growth. Altogether, our findings establish *H. pseudoflava* as a promising platform organism for sustainable syngas-based bioprocesses and provide a foundation for future metabolic and process engineering efforts toward bioplastic or biofuel production within a circular economy framework.

## 2 Materials and methods

### 2.1 Strain

All experiments were carried out with *H. pseudoflava* DSM1084 (Zavarzin and Nozhevnikova, 1977), obtained from DSMZ. The lyophilized strain was reactivated by transferring it into 50 mL of carbon monoxide oxidizer medium (CMOM) enriched with sodium acetate (3.0 g L^−1^) and subsequently incubated aerobically in shake flasks at 30 °C.

### 2.2 Medium composition

Unless mentioned otherwise, CMOM was used for bottle trials, precultures, and bioreactor Cultivations 1 and 2 (Grenz et al., 2019). The medium was prepared in four separate portions in order to avoid salt precipitation during autoclaving: Bottle 1 contained Na_2_HPO_4_ x 12 H_2_O (45 g L^−1^) and KH_2_PO_4_ (7.5 g L^−1^), bottle 2 NH_4_Cl (15 g L^−1^) and MgSO_4_ x 7 H_2_O (2 g L^−1^); bottle 3 CaCl_2_ x 2 H_2_O (0.3 g L^−1^) and ferric ammonium citrate (0.18 g L^−1^), and bottle 4 sodium bicarbonate (10 g L^−1^). The pH in all bottles was adjusted to 7 with 2 M NaOH or 2 M H_2_SO_4_ before autoclaving. After autoclaving, 100 mL of each solution was mixed with 1 mL of trace element solution 1 (TES1) and 599 mL of sterilized demineralized water. TES1 contained ZnSO_4_ x 7 H_2_O (0.1 g L^−1^), MnCl_2_ x 4 H_2_O (0.03 g L^−1^), H_3_BO_3_ (0.3 g L^−1^), CoCl_2_ x 6 H_2_O (0.2 g L^−1^), CuCl_2_ x 2 H_2_O (0.01 g L^−1^), NiCl_2_ x 6 H_2_O (0.02 g L^−1^), and Na_2_MoO_4_ x 2 H_2_O (0.03 g L^−1^). It was filter sterilized (0.22 µm bottle-top filter, polysulfone, VWR, U.S.A.) and stored at 4 °C.

For the bioreactor cultivations 3 and 4, an optimized medium (OCMOM) was used, which was composed of Na_2_HPO_4_ x 12 H_2_O (4.5 g L^−1^), KH_2_PO_4_ (3 g L^−1^), NH_4_Cl (6.5 g L^−1^), MgSO_4_ x 7 H_2_O (0.24 g L^−1^), CaCl_2_ x 2 H_2_O (0.031 g L^−1^) and ferric ammonium citrate (0.119 g L^−1^). The trace element solution 2 (TES2) contained ZnSO_4_ x 7 H_2_O (6.8 g L^−1^), MnCl_2_ x 4 H_2_O (0.037 g L^−1^), H_3_BO_3_ (0.3 g L^−1^), CoCl_2_ x 6 H_2_O (0.56 g L^−1^), CuCl_2_ x 2 H_2_O (0.097 g L^−1^), NiCl_2_ x 6 H_2_O (0.14 g L^−1^) and Na_2_MoO_4_ x 2 H_2_O (1.1 g L^−1^), and 10 mL of it was added per liter of medium unless mentioned otherwise.

### 2.3 Culture conditions

Cryostocks were prepared by growing *H. pseudoflava* into the exponential growth phase on CMOM and then mixing an aliquot of the culture with glycerol at a ratio of 5:1 (v v^−1^). The strain was stored as a 1.2 mL cryostock culture in cryotubes at −80 °C.

Preculture and bottle cultivations were performed in 100 mL gas tight bottles (clear glass injection bottle, Glas Artikel, Köln, Germany) containing 29 mL of CMOM, to which 1 mL of a cryo-stock culture was added. Cultures were incubated for 3 days with orbital shaking (120 rpm, 50 mm amplitude) at 30 °C. The gas mixture in the head space was flushed via needles piercing the bottle cap for 5 min once a day. The gas flowrate of 500 mLmin^−1^ was set with the mass flow controllers of the bioreactor as described below. In a standard cultivation, the first preculture was incubated for 3 days, then an aliquot of 1 mL was used to inoculate a second preculture which grew for 5 days. This procedure was implemented to ensure depletion of the glycerol remaining from the original frozen stock. From the second preculture, experiments were started, and the ensuing cultivation was carried out as long as the cell mass concentration kept increasing.

Bioreactor cultivations were carried out at 30 °C in a 3.6 L stirred bioreactor (Labfors 5, Infors HT AG, Bottmingen, Switzerland) with a working volume of 2 L under batch conditions. The vessel was equipped with 3 Rushton turbines and 3 baffles. The gas mixture was continuously sparged into the reactor through a porous sparger. The mixture was prepared in house from pure gases (PanGas, Lucerne, Switzerland, with purity >99.9%) and set with mass flow controllers ranging between 20% and 40% (v v^−1^) for CO, 2 and 4% for O_2_, 0% and 10% for CO_2_, and 40% for H_2_. The total gas flow rate was between 0.5 and 2 L min^−1^. These flowrates were chosen based on the range of syngas compositions and on the analysis performed in literature (Grenz et al., 2019).

### 2.4 Analytical methods

The pH in the reactor was determined inline with a pH probe (Hamilton, Bonaduz, Switzerland) and maintained at 7 by the automatic addition of aqueous H_2_SO_4_ (2 M) or NaOH (5 M). The concentrations of dissolved O_2_ and CO_2_ were measured online by two dedicated probes (O_2_: Hamilton, optical sensor with a measurement range of dissolved O_2_ of 0–21 mg L^−1^ in these conditions; CO_2_: Mettler Toledo, potentiometric Severinghaus sensor with a measurement range of pCO_2_ of 10–1000 hPa, equivalent to a concentration range between 1.4 mg L^−1^ and 1.4 g L^−1^ in these conditions).

During bioreactor cultivation, the composition of the gas at the reactor inlet and outlet were measured by a quadrupole mass spectrometer (MS) (Hiden Analytical QIC BioStream, Warrington, U.K.). The flowrates of the gas inlet and outlet were set to a maximum value of 2 mL min^−1^. The flowrates of the separate gas components were calculated by measuring the total flowrates and the respective gas compositions at the in- and outlet. H_2_ and CO concentrations were measured, but the differences in the concentrations between the gas inlet and outlet were too small compared to the actual concentration of these gases to be reliably quantified. The specific uptake rate of gas component E ( $q_{E}$ ) was computed using the growth yield ( $Y_{X/E}$ ) in the following equations (Equation 1) and (Equation 2).

$$q_{E}=\frac{\mu_{OD600}}{Y_{X/E}}\;\left[g\;g^{-1}h^{-1}\right]$$ (1)

with

$$Y_{X/E}=\frac{\int \left(F_{E}^{in}-F_{E}^{out}\right)dt}{DCW}\;\left[g\;g^{-1}\right]$$ (2)

and $F_{E}^{in/out}$ : Flowrate of the gas component at in- and outlet

The increase in biomass was quantified by two different methods. First, the optical density was measured with a spectrophotometer (Biochrom Ultrospec 2100 pro, Holliston, U.S.A.) at 600 nm (OD_600_), which was also used for the determination of the specific growth rates. During bottle cultivations, only OD_600_ measurements were performed. In general, we observed linear growth in time in bottle experiments. To obtain an estimate for the initial specific growth rate in these experiments, we assumed exponential growth between the two first OD_600_ measurements at time points t_1_ and t_2_ (Equation 3) and the specific growth rate was calculated using least-square regression on at least three data points on the linear part when plotting the natural logarithm of the OD_600_ against time (Equation 4).

$$\mu_{OD600}=\frac{\ln\left(OD_{600}\left(t_{2}\right)\right)-\ln\left(OD_{600}\left(t_{1}\right)\right)}{t_{2}-t_{1}}\;\left[h^{-1}\right]$$ (3)

$$\mu_{OD600}=\frac{Cov\left(t,\ln\left(OD_{600}\right)\right)}{Var\left(t\right)}\;\left[h^{-1}\right]$$ (4)

with $Cov\left(t,\ln\left(OD_{600}\right)\right)$ : Covariance of t and $\ln\left(OD_{600}\right)$

and $Var\left(t\right)$ : Variance of t .

Second, the dry cell weight (DCW) concentration was determined from triplicates of 4 mL of sample volume. For this, 2 mL Eppendorf reaction tubes were dried at 100 °C for 24 h and weighed. A sample was taken, introduced into an Eppendorf tube, centrifuged at 11000 *g* at 4 °C for 10 min, and the supernatant discarded. Then, another 2 mL of sample was introduced in the same Eppendorf tube and the procedure repeated. The pellets were resuspended in 1.5 mL of aqueous NaCl solution (0.9% w w^−1^), centrifuged again, and the supernatant was discarded. The pellets were dried at 100 °C for 24 h, and the difference in masses before and after the procedure allowed calculating the biomass concentration.

To measure the ammonium concentration in the culture broth, an assay kit (Spectroquant kit, Merck) was used. A portion of the samples (ca. 10 mL) was centrifuged at 9400 *g* at 4 °C for 10 min. The supernatant was then diluted between 5 and 2000 times. From this diluted solution, the assay kit was used according to the instructions of the manufacturer.

During bioreactor cultivations, the concentrations of various metal ions in the medium were determined by inductively coupled plasma (ICP) analysis (Agilent 5900 optical emission spectrometer, Santa Clara, U.S.A.). Aliquots of 20 mL were taken from the culture and centrifuged at 9000 *g* for 10 min. The supernatant was removed, acidified by the addition of HNO_3_ (99.9%) to a pH lower than 2, and ICP measurements were performed. The biomass yield coefficient for each metal and for nitrogen was computed from the ratio of DCW produced per mass of element consumed during the exponential growth phase.

### 2.5 Design of experiment and model scoring

For the optimization of the gas and medium compositions, cultivations in gas tight bottles were performed in parallel. To reduce the number of experiments a 2^4–1^ fractional factorial design was developed, meaning that each parameter can adopt 2 possible values and 4 parameters were analyzed which were in our case the molar percentage of O_2_, CO, CO_2_, and H_2_ in the gas fed. A fraction, here 50%, of all the 2^4^ possible combinations were tested, resulting in a total of 8 experiments repeated 3 times. Different models were compared to correlate specific growth rate and the changes in concentrations of the different gas or liquid medium compositions: linear, linear with interactions, quadratic and quadratic with interactions. The quality of the performance of the different models in correlation was scored based on the *p*-value of the lack of fit for the entire model and on the *p*-values obtained for each parameter. The software MATLAB R2020a (MathWorks, Natick, MA, U.S.A.) was used to develop and analyze the results.

## 3 Results and discussion

### 3.1 Effect of the gas composition on the specific growth rate of *H. pseudoflava*

**FIGURE 1:**
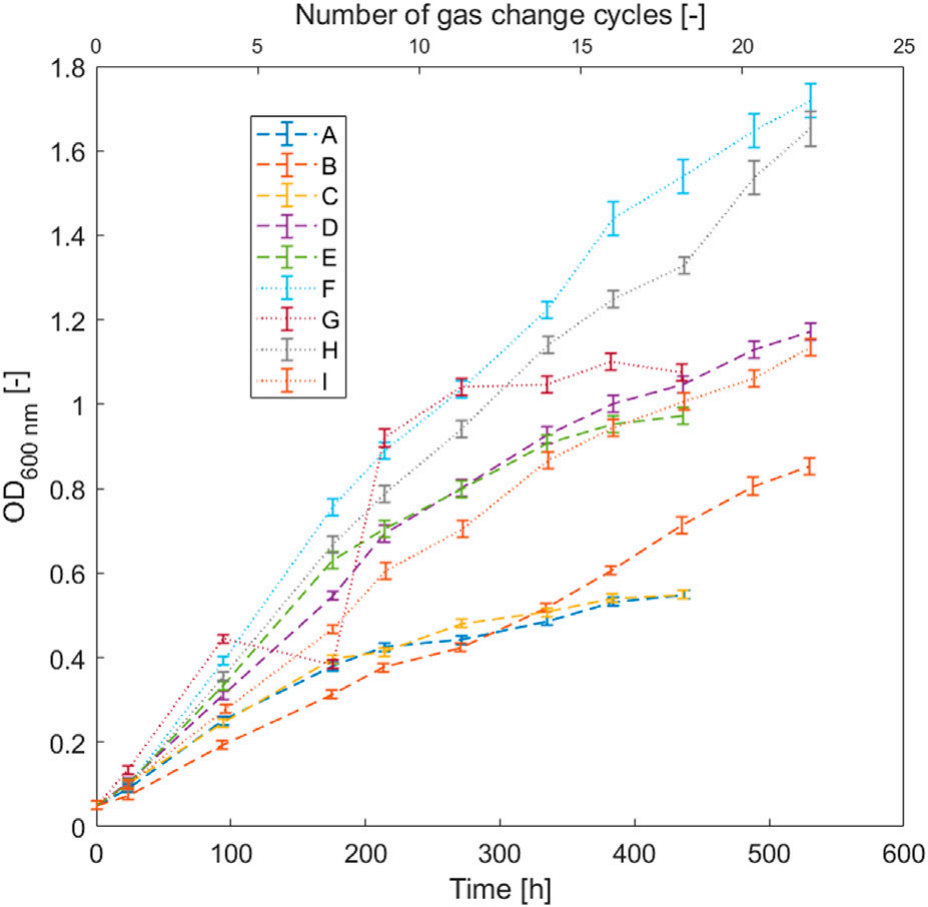
Effect of the gas composition on the growth of *H. pseudoflava* with the following compositions tested: H_2_: 20%–40%, CO: 20%–40%, CO_2_: 0%–10%, O_2_: 2%–4%, and N_2_: difference to 100%. The error bars represent the error on the spectrophotometer measurement multiplied by the dilution.

First, we optimized the growth rate of *H. pseudoflava* by varying the composition of the supplied gas while using CMOM liquid medium, which had also been used by Grenz et al. (2019) before. To efficiently identify an optimal gas composition, a DoE approach was applied (see Table 1) based on the gas composition reported by Grenz et al. (2019). During this study, the effects on the specific growth rate, biomass production, the volumetric CO consumption and CO_2_ production were assessed (see Figure 1).

**TABLE 1:** Gas composition used in the different bottles used for the cultivation of *H. pseudoflava* with gas exchanged each day.

Bottle	Gas composition [%]				
	H_2_	CO	CO_2_	O_2_	N_2_
A	20	20	0	2	58
B	20	40	10	2	28
C	40	40	0	2	18
D	40	20	10	2	28
E	20	40	0	4	36
F	20	20	10	4	46
G	40	20	0	4	36
H	40	40	10	4	6

It was found that biomass increase (OD_600_) followed a linear trend over the first hundred hours after inoculation and continued until 500 h for most cultures (Figure 1). A critical factor influencing the growth was the O_2_ concentration, because the highest biomass concentrations and fastest growth were obtained for cultures with 4% O_2_. However, linear growth is in general a strong indicator for a limited gas-liquid mass transfer, which can be reduced in well-stirred bioreactors with a high gas transfer efficiency and with a continuous feed of gases (Klöckner et al., 2013). In fact, bottle as well as shake flask cultivations have low gas transfer coefficients (k_L_a) which was estimated to be around 5 to 15 h^-1^ with a shaking rate of 150 rpm (Schiefelbein et al., 2013), while a value of 206 h^-1^ was found in the 3.6 L bioreactor. Based on these results, the gas transfer was the growth limiting factor (more details are given in the supplementary materials, see Supplementary Tables S1–S3).

An ANOVA analysis was performed to quantify the impact of the transfer of each gas component on the specific growth rate (Supplementary Table S1). In a first analysis including H_2_, CO, CO_2_, and O_2_, the model assuming a simple linear relationship between the different gas-components and growth showed the best performance (coefficient of determination 89.6%, *p*-value of the lack of fit was 38.8%), which confirmed that a simple linear model fitted best the data. This is consistent with the idea that the mass transfer of the different gas components was growth limiting in these experiments. However, a high *p*-value for the coefficient quantifying the influence of the CO_2_ concentration suggested a negligible influence of this component on growth. The ANOVA analysis was repeated without considering the CO_2_ concentration, and the coefficient of determination remained high (89.6%) but the *p*-value of the lack of fit increased to 52.0%, suggesting that the increase in growth had a stronger relationship with the model parameters for H_2_, CO, and O_2_ than for CO_2_ (Supplementary Table S4). Hence, the model developed without CO_2_ as parameter represented more accurately the relationship between the composition of the gas phase and the specific growth rate.

Analyses of the different gas components (Supplementary Tables S2, S4) revealed that increasing O_2_ and H_2_ concentrations in the gas mix had a positive impact on the specific growth rate.

In contrast, an increased CO fraction exhibited a negative effect. The highest specific growth rate was 0.042 h^−1^, achieved in an atmosphere with the highest fraction of H_2_ (40%) and O_2_ (4%) and the lowest fraction of CO (20%). This gas mixture was adopted for further experiments. CO_2_ was no longer added to the gas mixture because it had shown only a minimal impact.

### 3.2 Growth with optimized gas composition and transfer

To validate the findings of the bottle cultures, we used the optimized gas composition in a bioreactor.

It should be noted that the approximate specific growth rate obtained in small scale cultivations with shaken bottles (Table 1) was lower (µ = 0.021 h^−1^) than in cultivations conducted under the same gas composition as described by Grenz et al (2019) (µ = 0.06 h^−1^). This observation can be explained by the superior mass transfer coefficient from the gas to the liquid phase in a stirred tank bioreactor (Klöckner et al., 2013). As the solubilities in aqueous media for all three gas components (H_2_, CO, and O_2_) are very small (27 mg L^−1^ for CO, 1.57 mg L^−1^ for H_2_, and 42 mg L^−1^ for O_2_, all values for partial pressures of each gas of 1 bar at 25 °C (Sander, 2023)), stirring can be expected to play a critical role for adding and removing the gas components from the liquid medium. Hence, the optimal gas composition found in small-scale bottle experiments with repeated gas exchange was next tested under conditions of constant supply in a benchtop-scale bioreactor which allowed for improved mass transfer, off-gas analysis, and a quantification of the CO_2_ production. Two cultivations were performed: Cultivation 1 with the gas composition used by Grenz et al. (2019) (40% H_2_, 40% CO, 10% CO_2_, 2% O_2_) and Cultivation 2 with the composition identified as optimal in the experiments conducted here (40% H_2_, 20% CO, 0% CO_2_, 4% O_2_). The CO consumption is presented in the supplement (Supplementary Figure S2).

In contrast to the experiments carried out in bottles, we observed exponential growth in Cultivations 1 and 2, indicating that for much longer periods overall mass transfer for gas substrates was no longer a limiting factor.

**FIGURE 2:**
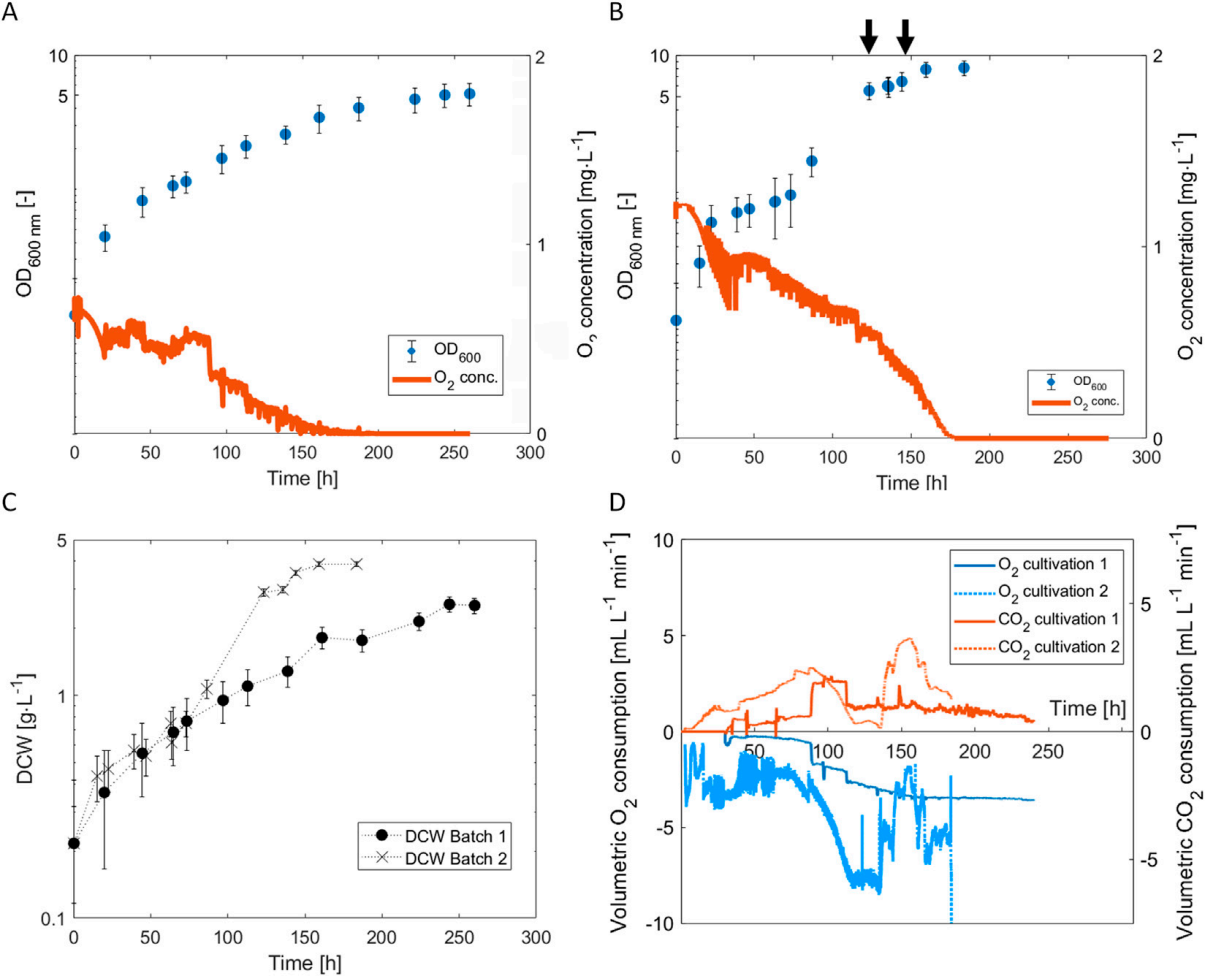
Growth of *H. pseudoflava* with two different constant gas supplies in 2 L of CMOM. **(A)** Development of the optical density at 600 nm and the dissolved O_2_ concentration in the medium with a gas composition of 40% H_2_, 40% CO, 10% CO_2_, 2% O_2_ (Cultivation 1); **(B)** 40% H_2_, 20% CO, 0% CO_2_, 4% O_2_ (Cultivation 2). The arrows (horizontal axis) indicate the addition of 2 mL of trace element solution TES; **(C)** Time course of the DCW concentration in Cultivations 1 and 2; **(D)** Time course of the gas consumption and production in Cultivations 1 and 2. The error bars represent the error on the spectrophotometer measurement multiplied by the dilution.

In Cultivation 1, we observed a specific growth rate of 0.047 h^−1^ ± 0.010 h^-1^ based on three OD_600_ measurements at around 40 h. Later, the specific growth rate decreased again. O_2_ levels reached 0 mg L^−1^ at around 200 h. When the O_2_ concentration decreased below 0.2 mg L^−1^ (after 150 h), specific growth rate decreased from 0.02 h^−1^ to 0.006 h^−1^, indicating again an O_2_ gas transfer limitation (Figure 2A).

In case of Cultivation 2 (Figure 2B), a specific growth rate of 0.072 h^−1^ ± 0.006 h^−1^ based on OD_600_ was calculated for the first 30 h. With the modified gas composition, the strain showed an increase in µ_max_ by 50% (Figure 2C). Notably, the specific growth rate sharply decreased in Cultivation 2 after 30 h, which was interpreted as a nutrient limitation in the liquid medium, as the O_2_ concentration was still high (>0.5 mg L^−1^). We reasoned that this limitation was most likely due to one of the trace elements. Indeed, the addition of 1 mL L^−1^ of trace element solution TES1 at t = 63 h resulted in an increase of the specific growth rate (from around 0 h^-1^ to 0.04 h^-1^ ± 0.002 h^−1^) until the dissolved O_2_ concentration in the medium dropped to 0 mg L^−1^ after 170 h of cultivation. However, the specific growth rate did not increase further to the initial value of the batch, even though the TES concentration in the medium was comparable to its initial concentration. A second addition of TES1 after 130 h (dissolved O_2_ concentration 0 mg L^−1^) did not trigger any bacterial growth in Cultivation 2 suggesting that another medium component restricted growth (see Section 3.3).

An important additional aspect of these experiments was to investigate to what extent an increase in specific growth rate would translate into enhanced CO_2_ production, which was assessed with off-gas mass spectrometry (Figure 2D). In Cultivation 1, CO_2_ production increased after 90 h and decreased again after 120 h. The maximal CO_2_ volumetric formation rate obtained was 2.5 mL L^−1^ min^−1^ at t = 160 h. In Cultivation 2, the CO_2_ production increased for around 80 h, then stopped between 110 h and 140 h, and then increased again after the second addition of trace elements and reached a maximum with a formation rate of 4 mL min^−1^ L^−1^. Interestingly, the maximum specific CO_2_ production was approximately constant in Cultivations 1 and 2 (1.9 mL min^−1^ g^−1^(DCW) for Cultivation 1 and 2.1 mL min^−1^ g^−1^ (DCW) for cultivation 2). However, with the increased cell dry weight concentration in Cultivation 2, also the maximal volumetric CO_2_ increased, from 2.5 mL L^-1^ min^−1^ (Cultivation 1) to 4 mL L^−1^ min^−1^ (Cultivation 2).

In conclusion, the optimized gas composition led to an increase in specific growth rate and an increase by 60% of the absolute CO_2_ output rate (from 5 mL min^−1^ to 8 mL min^−1^) and a higher maximal DCW.

### 3.3 Effect of the liquid medium composition on the specific growth rate

Having optimized the gas composition, we next optimized the composition of the liquid medium with respect to trace elements using ICP analytics. Throughout Cultivation 1, all metals originally supplied in the trace element solution were still present in the culture broth, whereas Fe was totally consumed after less than 15 h and Cu and Mo after 160 h. However, the specific growth rate had decelerated already after 45 h of cultivation, probably due to gas transfer limitations. During Cultivation 2, growth stopped after 30 h of cultivation, suggesting that some elements provided by solution TES1 had been completely depleted already by that time. Indeed, injections of 2 mL of trace element solution reinitiated exponential growth for another 70 h after the first injection, and for 20 h after a second injection at cultivation time 135 h, presumably due to a higher cell density which led to a faster exhaustion of the trace element in question.

**FIGURE 3:**
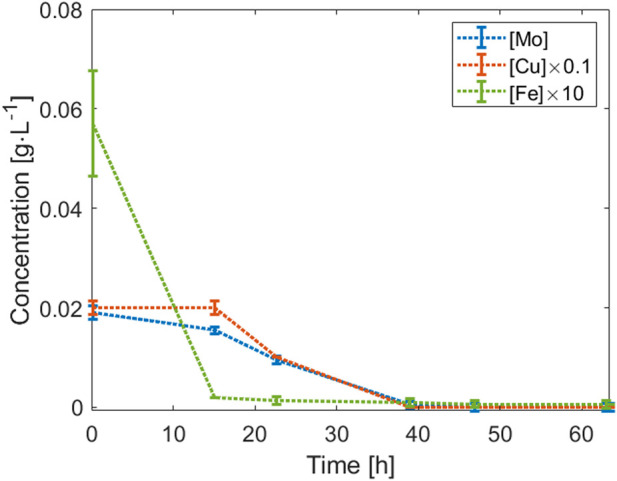
Time course of the Mo, Cu, and Fe concentration during the first 70 h of Cultivation 2 before the addition of trace element solution. The error bars represent the standard deviation of the concentration of the Mo, Cu, and Fe concentration (measurement performed two times for each element).

Put differently, at the latest after 40 h of cultivation in Cultivation 2, growth was multiple-nutrient limited (Figure 3) (Karmann et al., 2019). Interestingly, a concomitant change of the trace-element metabolism could have taken place which has already been reported previously (Mills, 1964; Merchant and Helmann, 2012).

In view of medium optimization, we calculated the yield coefficients for the three critical metals and other potentially important metal species, and nitrogen in the CMOM liquid medium. A comparison with average yield coefficients obtained for different bacteria cultivated under different conditions (heterotrophic and chemoautotrophic conditions) was made (Table 2) (time course of the Co, Mn, Ni, Zn, Ca, and Mg and detailed yield coefficients obtained for each cultivation are presented in Supplementary Figure S1; Supplementary Table S5). Differences between the yield coefficients obtained and the reference ones could be caused by the cultivation conditions. A comparison of the values for Ca, Cu, and Fe was conducted to identify potential causes of the observed deviations. In general, the values obtained for our cultivations were in the same order of magnitude as the ones mentioned in literature, even if the values obtained were not from strains able to grow on syngas, with the exception of Ca, which seemed to be less required by *H. pseudoflava*. It has been shown that Ca is required by cells for motility, maintenance of cell structure, transport, and cell differentiation processes (Dominguez, 2004). Less prominent yet still remarkable differences between literature values and measured yield coefficients include Cu and Fe. Cu is a key metal for the enzyme CO dehydrogenase (Hänzelmann et al., 2000; Hille, Dingwall, and Wilcoxen, 2015) and thus may explain the decreased biomass yield coefficient (i.e., higher Cu requirements for growth on CO). As mentioned previously, the Fe containing CO dehydrogenase is still functional at low Fe concentration and under aerobic conditions and thus may explain the higher biomass yield coefficient obtained (Hänzelmann et al., 2000). Importantly, and unlike in anaerobic bacteria, the CO dehydrogenase enzyme of carboxydotrophic bacteria contains less Fe (with two subunits containing Fe-S clusters instead of four), reducing overall Fe needs (Ruickoldt et al., 2022; Hille, Dingwall, and Wilcoxen, 2015), explaining the higher yield coefficient obtained than the one found in literature (compare Table 2). Mo was also consumed rapidly, decreasing to 0 g L^−1^ after only 40 h of cultivation. Mo is a key element for *H. pseudoflava* and carboxidotrophs in general. In fact, aerobic CO dehydrogenase is composed of Mo (Hänzelmann et al., 2000).

**TABLE 2:** Biomass yield coefficients for different medium components.

Element (E)	Ca	Co	Cu	Fe	Mg	Mo	N	Ni	Zn
Y_X/E_ [g g^-1^]	1.7 × 10^3^	7.2 × 10^4^	2.8 × 10^4^	7.4 × 10^2^	4.2 × 10^2^	2.3 × 10^3^	6.8	2.8 × 10^4^	6.5 × 10^3^
Approx. Y_X/E_ based on literature (Pirt, 1975; Egli, 2009) [g g^-1^]	10^2^	10^5^	10^5^	200	200	-	8	-	10^4^

Next, we took advantage of the data on yield coefficients (Table 2) and optimized the concentrations of the nutrients in the new medium OCMOM to the physiological needs of *H. pseudoflava*, aiming at a final biomass concentration of 10 g L^−1^. Specifically, the concentrations for Ca, Mo, Fe, Cu, and N were increased as follows (relative to CMOM): Ca 1.04-fold, Mo 3500-fold, Fe 57-fold, N 36-fold, and Cu 10-fold. An increase of the Fe, Cu, and Mo concentration in the medium was needed as they are important components in the CO-dehydrogenase. Attempts to improve OCMOM for even higher DCW concentrations remained unsuccessful as at higher salt concentrations precipitation occurred during autoclaving which we could not resolve by splitting and re-uniting medium components as we had done for CMOM medium.

**FIGURE 4:**
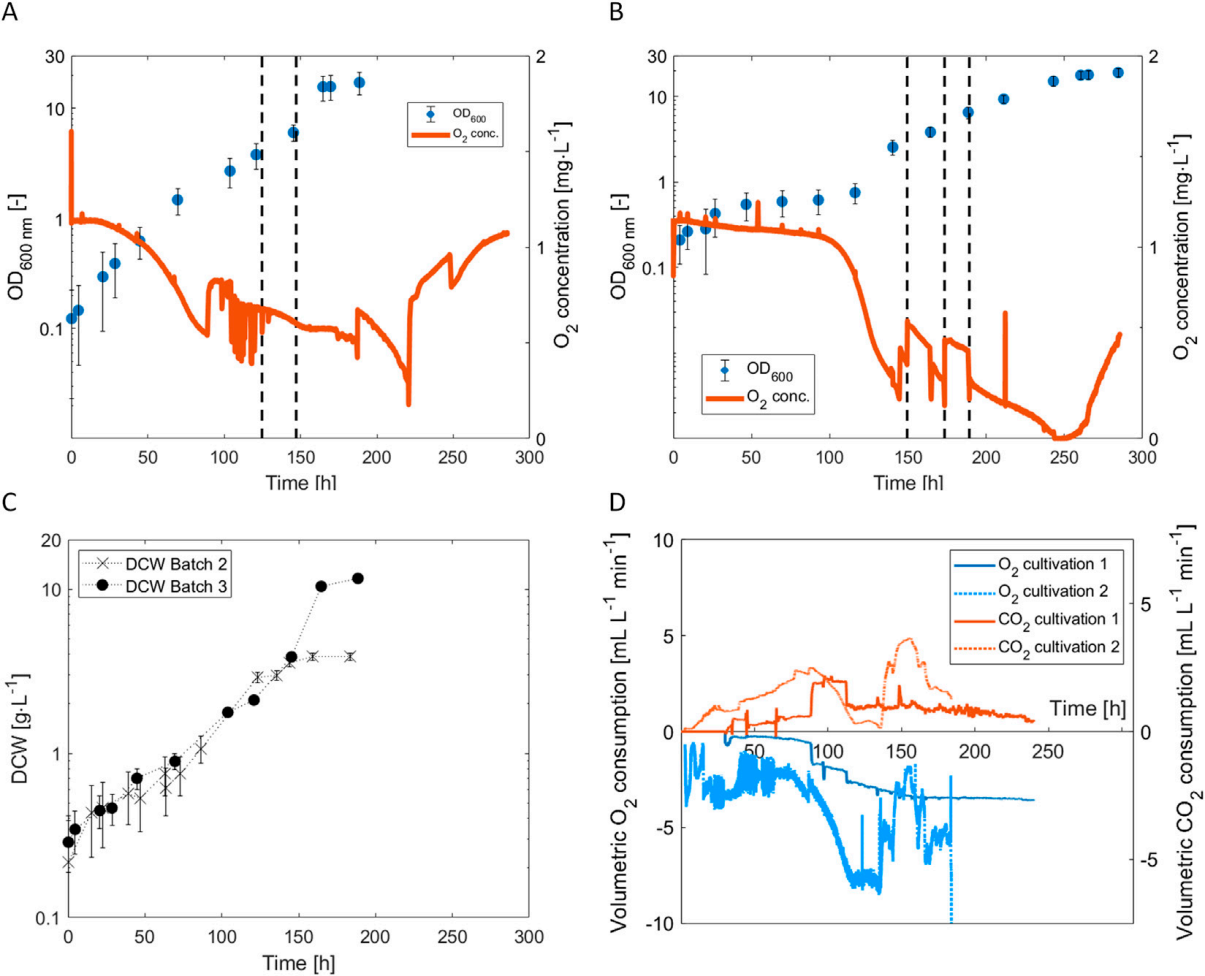
Bioreactor cultivations of *H. pseudoflava* with optimized gas (40% H_2_, 20% CO, 0% CO_2_, 4% O_2_) and liquid medium compositions (OCMOM). **(A)** Time course of OD_600_ and the concentration of dissolved O_2_ during Cultivation 3, the dotted lines stand for the increase of the gas flowrate from 0.5 L min^−1^ to 1 L min^−1^ at 125 h and 1.5 L min^−1^ at 147 h. **(B)** Time course of the optical density at 600 nm and the concentration of dissolved O_2_ during Cultivation 4, the dotted lines stand for the increase of the gas mixture flowrate from 0.5 L min^−1^ to 1 L min^−1^ at 149.5 h, 1.5 L min^−1^ at 173 h, and 1 L min^−1^ at 189 h; **(C)** Time course of the DCW during cultivation 2 (optimized gas only) and cultivation 3 (optimized gas and OCMOM); **(D)** Time course of the gas consumption and production during Cultivation 4 (not quantified during Cultivation 3). The error bars represent the error on the spectrophotometer measurement multiplied by the dilution.

Next, OCMOM was tested at bench reactor scale. The optimized gas composition (40% H_2_, 20% CO, 0% CO_2_, and 4% O_2_) was used again with the same gas flowrate at the beginning but increasing throughout the fermentation (see Figure 4). The fermentation was performed twice (Cultivations 3 and 4). Their final DCW was 11.6 g L^−1^ for Cultivation 3 and 12.0 g L^−1^ for Cultivation 4, and therefore slightly higher than the targeted 10 g L^−1^. This suggests that the previous yield calculations were valid. In fact, the concentrations of the critical trace elements Mo, Fe as well as Zn were below the detection limit at the end of Cultivation 3.

We adapted the feeding strategy in view of the expected higher dry cell weight concentrations in Cultivations 3 and 4: During both cultivations, the gas flow rate was increased in increments of 0.5 L min^−1^ when the dissolved O_2_ decreased below 0.5 mg L^−1^. This allowed enhancing the O_2_ supply without entering the domain of explosive gas mixtures. In fact, the dissolved O_2_ concentration never decreased to zero in the course of Cultivation 3 and only briefly during Cultivation 4. The CO consumption is shown in the supplementary information (Supplementary Figure S2).

During Cultivations 3 and 4, the µ_max_ was 0.06 h^−1^ ± 0.007 h^−1^ (computed between 0 and 40 h) and was comparable to Cultivation 2. The medium composition did not have an impact on the µ_max_. In Cultivation 3, the specific growth rate remained constant during the first 70 hours (Figure 4A) while it decreased in Cultivation 4 between 40 h and 120 h and then increased again to the initial value (Figure 4B). This pause in the bacterial growth was observed only for Cultivation 4. As cultivation conditions and bioreactor settings were kept constant during the trials, the reason for this phase remained unclear.

The maximum specific volumetric CO_2_ production rate, recorded during Cultivation 4, was 7.5 mL L^−1^ min^−1^ at 30 °C and atmospheric pressure (Figure 4D). This increase was attributed to the higher dry cell weight (DCW) observed under these conditions (Figure 4C), which doubled compared to the initial value obtained using the gas and medium composition described by Grenz et al. (2019). After 149 h of cultivation, CO_2_ production decreased in Cultivation 4 (Figure 4D). This decrease was due to a higher dilution of the CO_2_ produced after increasing the total gas flowrate to avoid O_2_ limitation. During this study, gas and medium compositions improvements were performed on the wild strain while Grenz et al. were cultivating a recombinant one to produce the chemical (E)-α-bisabolene (Grenz et al., 2019). Moreover, the ratio gas to liquid volume was higher during our experiments with a value of 0.3 for bottle trials (0.1 for Grenz et al.) and a ratio working volume and bioreactor volume of 0.7 (0.5 for Grenz et al.) which can decrease the mass transfer rate of the gases. However, the stirring system in this study was composed of 3 Rushton turbines (2 for Grenz et al.) and the gas was sparged within the medium directly [not described by Grenz et al. (2019)], improving the mass transfer rate.

In view of selective CO-depletion of syngas, it should be noted that CO was not completely consumed in any of the cultivations, it was always still present in the gas outlet. However, strategies can be devised to further reduce the CO in the gas outlet, such as recycling the gas stream at the outlet with some addition of O_2_ or a specific dilution with an N_2_/O_2_ mixture to establish a simultaneous CO and O_2_ limitation. To remain below the lower flammable limit, the O_2_ concentration in the gas would need to be monitored and used to control the O_2_ addition to the recycled gas.

## 4 Conclusion

This study highlights the significant potential of *H. pseudoflava* for syngas valorization under aerobic conditions. By systematically optimizing both the gas and liquid medium compositions, we achieved substantial improvements in biomass yield and CO_2_ production. Specifically, an optimized gas mixture of 40% H_2_, 20% CO, and 4% O_2_ (with CO_2_ excluded) supported the highest specific growth rate of μ = 0.072 h^−1^ (μ = 0.06 h^−1^ in the work of Grenz et al. (2019)) reported so far for autotrophic growth of *H. pseudoflava* and enabled controlled, higher density growth in stirred tank bioreactors.

Further optimization of the liquid medium, based on trace element consumption analyses, allowed the formulation of OCMOM - a medium tailored to the physiological needs of *H. pseudoflava*. It supported dry cell weight concentrations exceeding 11 g L^−1^, along with a peak CO_2_ production rate of 7.5 mL L^−1^ min^−1^, more than double than achieved using previously reported conditions.

Altogether, our findings establish *H. pseudoflava* as a robust and scalable platform organism for syngas-based bioprocesses. The combination of high growth rates, efficient gas utilization, and compatibility with aerobic operation, positions this system as an attractive route for sustainable carbon recycling. In particular, the demonstrated performance under industrially relevant conditions underscores the feasibility of translating this process toward bioplastic and biofuel production, supporting broader efforts to reduce reliance on fossil-derived feedstocks and advance circular bioeconomy strategies.

## Data Availability

The raw data supporting the conclusions of this article will be made available by the authors, without undue reservation.
