# Supplementary material for: Growth of Hydrogenophaga pseudoflava on syngas: design of gas and liquid medium composition based on elemental yield coefficients
Source: Front Bioeng Biotechnol. 2026 Jan 7;13:1727931. doi: 10.3389/fbioe.2025.1727931 (PMC12818879; doi:10.3389/fbioe.2025.1727931)
Supplement: Supplementary file 1 [file Supplementaryfile1.docx]

Supplementary Material

Growth of *Hydrogenophaga pseudoflava* on syngas: Design of gas and liquid medium composition based on elemental yield coefficients

Florian Miserez^1,2^, Sven Panke^1^, Manfred Zinn^2*^

# Effect of the gas composition on the specific growth rate

To optimize the gas composition, analysis of the effect of each gas component was determined by performing a DoE with fractional factorial design (2^4-1^). This was done to test different combinations of gas and also to reduce the number of experiments. The results are summarized in the ANOVA table (Supplementary Table 1) for the linear model without interactions (p-value of the model: 0.00002, R-squared: 0.896), Supplementary Table 2 for the linear model with interactions (*p*-value of the model: 0.0006, R-squared: 0.927) and Supplementary Table 3 for the quadratic model (*p*-value of the model: 0.0006, R-squared: 0.927).

Supplementary Table 1. ANOVA table of the model $\mathbf{y=}\mathbf{a}_{\mathbf{0}}\mathbf{+}\mathbf{a}_{\mathbf{1}}\boldsymbol{\cdot}\mathbf{x}_{\mathbf{1}}\mathbf{+}\mathbf{a}_{\mathbf{2}}\boldsymbol{\cdot}\mathbf{x}_{\mathbf{2}}\mathbf{+}\mathbf{a}_{\mathbf{3}}\boldsymbol{\cdot}\mathbf{x}_{\mathbf{3}}\mathbf{+}\mathbf{a}_{\mathbf{4}}\boldsymbol{\cdot}\mathbf{x}_{\mathbf{4}}$ with y the specific growth rate, a_0_ the coefficient of the constant effect; a_1_, a_2_, a_3_, a_4_ the coefficient of the main effect of the percentage of respectively O_2_, H_2_, CO_2_, and CO, y the response, x_1_, x_2_, x_3_, x_4_ the variables of the model for O_2_, H_2_, CO_2_, and CO, respectively.

| **Effect** | **Estimate** | **SS**^1^ | **DF**^2^ | **MS**^3^ | **F**^4^ | ***p*-value** |
| --- | --- | --- | --- | --- | --- | --- |
| a_0_ | 0.018300 |  | 1 |  |  | 1.8×10^-16^ |
| a_1_ (O_2_) | 0.002000 | 6.7×10^-5^ | 1 | 6.7×10^-5^ | 76.92 | 2.7×10^-6^ |
| a_2_ (H_2_) | 0.000700 | 8.6×10^-6^ | 1 | 8.6×10^-6^ | 9.85 | 9.4×10^-3^ |
| a_3_ (CO_2_) | -0.000056 | 5.1×10^-8^ | 1 | 5.1×10^-8^ | 0.06 | 8.1×10^-1^ |
| a_4_ (CO) | -0.000600 | 6.6×10^-6^ | 1 | 6.6×10^-6^ | 7.63 | 1.8×10^-2^ |
| Residual |  | 9.6×10^-6^ | 11 | 8.7×10^-7^ |  |  |

^1^ SS: Sum of squares

^2^ DF: Degrees of freedom

^3^ MS: Mean squares for each source, which is the ratio SS/Df

^4^ F-statistic, which is the ratio of the mean squares

Supplementary Table 2. ANOVA table of the model $\mathbf{y=}\mathbf{a}_{\mathbf{0}}\mathbf{+}\mathbf{a}_{\mathbf{1}}\boldsymbol{\cdot}\mathbf{x}_{\mathbf{1}}\mathbf{+}\mathbf{a}_{\mathbf{2}}\boldsymbol{\cdot}\mathbf{x}_{\mathbf{2}}\mathbf{+}\mathbf{a}_{\mathbf{3}}\boldsymbol{\cdot}\mathbf{x}_{\mathbf{3}}\mathbf{+}\mathbf{a}_{\mathbf{4}}\boldsymbol{\cdot}\mathbf{x}_{\mathbf{4}}\mathbf{+}\mathbf{a}_{\mathbf{12}}\boldsymbol{\cdot}\mathbf{x}_{\mathbf{1}}\boldsymbol{\cdot}\mathbf{x}_{\mathbf{2}}\mathbf{+}\mathbf{a}_{\mathbf{13}}\boldsymbol{\cdot}\mathbf{x}_{\mathbf{1}}\boldsymbol{\cdot}\mathbf{x}_{\mathbf{3}}\mathbf{+}\mathbf{a}_{\mathbf{14}}\boldsymbol{\cdot}\mathbf{x}_{\mathbf{1}}\boldsymbol{\cdot}\mathbf{x}_{\mathbf{4}}\mathbf{+}\mathbf{a}_{\mathbf{23}}\boldsymbol{\cdot}\mathbf{x}_{\mathbf{2}}\boldsymbol{\cdot}\mathbf{x}_{\mathbf{3}}\mathbf{+}\mathbf{a}_{\mathbf{24}}\boldsymbol{\cdot}\mathbf{x}_{\mathbf{2}}\boldsymbol{\cdot}\mathbf{x}_{\mathbf{4}}\mathbf{+}\mathbf{a}_{\mathbf{34}}\boldsymbol{\cdot}\mathbf{x}_{\mathbf{3}}\boldsymbol{\cdot}\mathbf{x}_{\mathbf{4}}$ with y the specific growth rate, a_0_ the coefficient of the constant effect; a_1_, a_2_, a_3_, a_4_ the coefficient of the main effect of the percentage of respectively O_2_, H_2_, CO_2_, and CO, y the response, x_1_, x_2_, x_3_, x_4_ the variables of the model for O_2_, H_2_, CO_2_, and CO, respectively.

| **Effect** | **Estimate** | **SS** | **DF** | **MS** | **F** | ***p*-value** |
| --- | --- | --- | --- | --- | --- | --- |
| a_0_ | 0.018300 |  | 1 |  |  | 1.8×10^-16^ |
| a_1_ (O_2_) | 0.002000 | 6.7×10^-5^ | 1 | 6.7×10^-5^ | 76.92 | 2.7×10^-6^ |
| a_2_ (H_2_) | 0.000700 | 8.6×10^-6^ | 1 | 8.6×10^-6^ | 9.85 | 9.4×10^-3^ |
| a_3_ (CO_2_) | -0.000056 | 5.1×10^-8^ | 1 | 5.1×10^-8^ | 0.06 | 8.1×10^-1^ |
| a_4_ (CO) | -0.000600 | 6.6×10^-6^ | 1 | 6.6×10^-6^ | 7.63 | 1.8×10^-2^ |
| a_12_ (O_2_:H_2_) | -0.000300 | 0 | 0 | - | - | - |
| a_13_ (O_2_:CO_2_) | -0.000200 | 0 | 0 | - | - | - |
| a_14_ (O_2_:CO) | -0.000200 | 0 | 0 | - | - | - |
| a_23_ (H_2_:CO_2_) | 0 | 0 | 0 | - | - | - |
| a_24_ (H_2_:CO) | 0 | 0 | 0 | - | - | - |
| a_34_ (CO_2_:CO) | 0 | 0 | 0 | - | - | - |
| Residual |  | 6.7×10^-6^ | 8 | 8.4×10^-7^ |  |  |

Supplementary Table 3. ANOVA table of the model $\mathbf{y=}\mathbf{a}_{\mathbf{0}}\mathbf{+}\mathbf{a}_{\mathbf{1}}\boldsymbol{\cdot}\mathbf{x}_{\mathbf{1}}\mathbf{+}\mathbf{a}_{\mathbf{2}}\boldsymbol{\cdot}\mathbf{x}_{\mathbf{2}}\mathbf{+}\mathbf{a}_{\mathbf{3}}\boldsymbol{\cdot}\mathbf{x}_{\mathbf{3}}\mathbf{+}\mathbf{a}_{\mathbf{4}}\boldsymbol{\cdot}\mathbf{x}_{\mathbf{4}}\mathbf{+}\mathbf{a}_{\mathbf{12}}\boldsymbol{\cdot}\mathbf{x}_{\mathbf{1}}\boldsymbol{\cdot}\mathbf{x}_{\mathbf{2}}\mathbf{+}\mathbf{a}_{\mathbf{13}}\boldsymbol{\cdot}\mathbf{x}_{\mathbf{1}}\boldsymbol{\cdot}\mathbf{x}_{\mathbf{3}}\mathbf{+}\mathbf{a}_{\mathbf{14}}\boldsymbol{\cdot}\mathbf{x}_{\mathbf{1}}\boldsymbol{\cdot}\mathbf{x}_{\mathbf{4}}\mathbf{+}\mathbf{a}_{\mathbf{23}}\boldsymbol{\cdot}\mathbf{x}_{\mathbf{2}}\boldsymbol{\cdot}\mathbf{x}_{\mathbf{3}}\mathbf{+}\mathbf{a}_{\mathbf{24}}\boldsymbol{\cdot}\mathbf{x}_{\mathbf{2}}\boldsymbol{\cdot}\mathbf{x}_{\mathbf{4}}\mathbf{+}\mathbf{a}_{\mathbf{34}}\boldsymbol{\cdot}\mathbf{x}_{\mathbf{3}}\boldsymbol{\cdot}\mathbf{x}_{\mathbf{4}}\mathbf{+}\mathbf{a}_{\mathbf{5}}\boldsymbol{\cdot}\mathbf{x}_{\mathbf{1}}^{\mathbf{2}}\mathbf{+}\mathbf{a}_{\mathbf{6}}\boldsymbol{\cdot}\mathbf{x}_{\mathbf{2}}^{\mathbf{2}}\mathbf{+}\mathbf{a}_{\mathbf{7}}\boldsymbol{\cdot}\mathbf{x}_{\mathbf{3}}^{\mathbf{2}}\mathbf{+}\mathbf{a}_{\mathbf{8}}\boldsymbol{\cdot}\mathbf{x}_{\mathbf{4}}^{\mathbf{2}}$ with y the specific growth rate, a_0_ the coefficient of the constant effect; a_1_, a_2_, a_3_, a_4_ the coefficient of the main effect of the percentage of respectively O_2_, H_2_, CO_2_, and CO, y the response, x_1_, x_2_, x_3_, x_4_ the variables of the model for O_2_, H_2_, CO_2_, and CO, respectively.

| **Effect** | **Estimate** | **SS** | **DF** | **MS** | **F** | ***p*-value** |
| --- | --- | --- | --- | --- | --- | --- |
| a_0_ | 0.018300 |  | 1 |  |  | 1.8×10^-16^ |
| a_1_ (O_2_) | 0.002000 | 6.7×10^-5^ | 1 | 6.7×10^-5^ | 76.92 | 2.7×10^-6^ |
| a_2_ (H_2_) | 0.000700 | 8.6×10^-6^ | 1 | 8.6×10^-6^ | 9.85 | 9.4×10^-3^ |
| a_3_ (CO_2_) | -0.000056 | 5.1×10^-8^ | 1 | 5.1×10^-8^ | 0.06 | 8.1×10^-1^ |
| a_4_ (CO) | -0.000600 | 6.6×10^-6^ | 1 | 6.6×10^-6^ | 7.63 | 1.8×10^-2^ |
| a_12_ (O_2_:H_2_) | -0.000300 | 0 | 0 | - | - | - |
| a_13_ (O_2_:CO_2_) | -0.000200 | 0 | 0 | - | - | - |
| a_14_ (O_2_:CO) | -0.000200 | 0 | 0 | - | - | - |
| a_23_ (H_2_:CO_2_) | 0 | 0 | 0 | - | - | - |
| a_24_ (H_2_:CO) | 0 | 0 | 0 | - | - | - |
| a_34_ (CO_2_:CO) | 0 | 0 | 0 | - | - | - |
| a_5_ | 0 | 0 | 0 | - | - | - |
| a_6_ | 0 | 0 | 0 | - | - | - |
| a_7_ | 0 | 0 | 0 | - | - | - |
| a_8_ | 0 | 0 | 0 | - | - | - |
| Residual |  | 6.7×10^-6^ | 8 | 8.4×10^-7^ |  |  |

Given that multiple parameters of the gas phase composition were changed concomitantly in this series of experiments, different statistical models were tested to describe the influence of gas components on the specific growth rate. Interestingly, a simple linear model gave the best fit to the data, quadratic parameters being at 0 for the quadratic model and 0 for the interaction parameters which is the value obtained for these parameters for the linear model with interaction and the quadratic one. An ANOVA analysis was performed to quantify the impact of each gas component on the specific growth rate (Supplementary Tables S1, S2, and S3). The coefficient of determination for the model implemented was 89.6 % and the *p*-value of the lack of fit was 0.00002, which confirmed the suitability of the linear model (*p*-values for the other model 30 times higher). Thus, a first model included the concentrations of the four gases O_2_, CO, CO_2_, and H_2_ as parameters and clearly showed that increasing O_2_ and H_2_ fractions have a positive impact on the specific growth rate. This is especially true for O_2_, with the strongest impact (around 10 times higher than H_2_). CO_2_ and especially CO have a negative effect on the specific growth rate. However, the high *p*-value of CO_2_ indicates a negligible correlation between its concentration and the specific growth rate. Thus, CO_2_ concentration was removed from the model (Supplementary Table 4).

Supplementary Table 4. Results obtained after removing CO_2_ from the model, ANOVA table of the model $\mathbf{y=}\mathbf{a}_{\mathbf{0}}\mathbf{+}\mathbf{a}_{\mathbf{1}}\boldsymbol{\cdot}\mathbf{x}_{\mathbf{1}}\mathbf{+}\mathbf{a}_{\mathbf{2}}\boldsymbol{\cdot}\mathbf{x}_{\mathbf{2}}\mathbf{+}\mathbf{a}_{\mathbf{3}}\boldsymbol{\cdot}\mathbf{x}_{\mathbf{3}}$ with a_0_ the coefficient of the constant effect; a_1_, a_2_, a_3_ the coefficient of the main effect of the percentage of respectively O_2_, H_2_ and CO, y the response, x_1_, x_2_, x_3_ the variables of the model for respectively O_2_, H_2_ and CO.

| **Effect** | **Estimate** | **SS** | **DF** | **MS** | **F** | ***p*-value** |
| --- | --- | --- | --- | --- | --- | --- |
| a_0_ | 0.0183 |  | 1 |  |  | 7.3×10^-18^ |
| a_1_ (O_2_) | 0.0020 | 6.7×10^-5^ | 1 | 6.7×10^-5^ | 83.4730 | 9.4×10^-7^ |
| a_2_ (H_2_) | 0.0007 | 8.6×10^-6^ | 1 | 8.6×10^-6^ | 10.6860 | 6.7×10^-3^ |
| a_3_ (CO) | -0.0006 | 6.6×10^-6^ | 1 | 6.6×10^-6^ | 8.2818 | 1.4×10^-7^ |
| Residual |  | 9.6×10^-6^ | 12 | 8.0×10^-7^ |  |  |

For this second linear model, a new ANOVA analysis was developed, and the results are summarized in Supplementary Table 4. The coefficients did not significantly change confirming that CO_2_ does not have an impact on the specific growth rate in these experiments. The coefficient of determination for the second model is still 89.6 % and the *p*-value of the lack of fit increases from 38.8 % to 52.0 %, supporting the notion that the concentration of carbon dioxide does indeed not have a significant effect on the specific growth rate. Like the previous model, O_2_ had the largest impact on the specific growth rate with the biggest estimated coefficient, followed by H_2_, both having a positive impact on the specific growth rate. CO showed a negative impact.

# Effect of the liquid medium composition


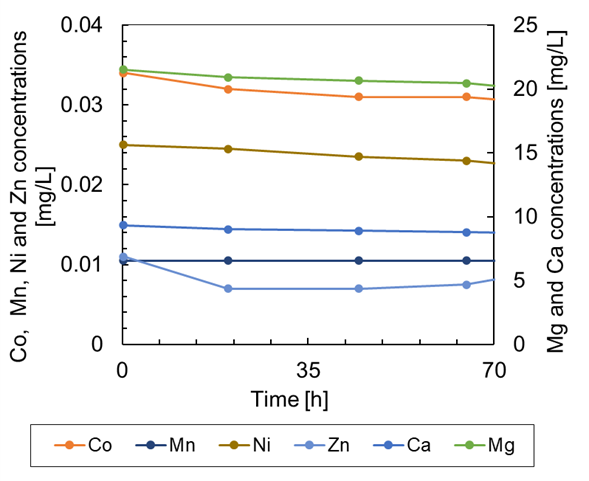


Supplementary Figure 1. Time course of the Co, Mn, Ni, Zn, Ca and Mg concentration during the first 70 h of Cultivation 1 (time period before addition of supplementary trace element solution).

After improving the gas composition, the medium composition was improved in order to increase bacterial growth and thus CO_2_ production. After performing Batches 1 and 2, it appeared that some elements were limiting the growth (Supplementary Figure 1). Given the concentration profiles of various medium components, the effect of the concentration of Cu, Mo, and Ca on the specific growth rate using DoE and bottle cultivations was analyzed with the previously determined optimal gas composition (40% H_2_, 20% CO, 0% CO_2_, 4% O_2_). These elements were chosen based on their total consumption during Batch 1 and 2 because aerobic CO-dehydrogenase is a Mo- and Cu-based enzyme. The elemental analysis in the culture supernatants yielded following concentrations for the batch cultures 1 and 2: Cu: 0.0587 and 0.176 mmol L^-1^, Mo: 0.124 and 0.372 mmol L^-1^, and Fe: 0.0687 and 0.206 mmol L^-1^ while the other medium compounds were as described in Material and Methods for the CMOM medium.

In order to improve the medium composition further, the biomass yield coefficients were computed during batches 1 and 2 by measuring the elemental concentrations using ICP (Supplementary Table 5).

Supplementary Table 5. Biomass yield coefficients of different medium elements (Y_X/E_) obtained during Batches 1 and 2 (Table 2 completed with values for sulfur).

| **Element (E)** | **Ca** | **Co** | **Cu** | **Fe** | **Mg** | **Mo** | **N** | **S** | **Zn** |
| --- | --- | --- | --- | --- | --- | --- | --- | --- | --- |
| Y_X/E_ batch 1  [g g^-1^] | 1.9×10^3^ | -^1)^ | 2.8×10^4^ | 1.1×10^3^ | 4.2×10^2^ | 2.3×10^4^ | 5.9 | 1.4×10^3^ | -^1)^ |
| Y_X/E_ batch 2  [g g^-1^] | 1.2×10^3^ | 7.2×10^4^ | -^1)^ | 3.9×10^2^ | -^1)^ | -^1)^ | 7.6 | 1.7×10^3^ | 6.5×10^3^ |
| Approx. Y_X/E_ based on literature (Pirt 1975; Egli 2009) [g g^-1^] | 10^2^ | 10^5^ | 10^5^ | 200 | 200 | -^2)^ | 8 | 10^2^ | 10^4^ |
| Concentration of E at the end of the cultivation batch 1 [mg L^-1^] | 9.7 | 0.028 | BQL^3)^ | 0.011 | 18.2 | BDL^4)^ | 176 | 2.4 | 0.01 |
| Concentration of E before addition of TES^5)^ batch 2 [mg L^-1^] | 5.5 | 0.020 | BDL^4)^ | BDL^4)^ | 8.7 | BDL^4)^ | BDL^4)^ | 1.3 | 0.009 |

^1)^ No linear correlation found between DCW and E concentration

^2)^ Not found in the literature

^3)^ BQL: Below the quantification limit

^4)^ BDL: Below the detection limit

^5)^ TES: Trace element solution

The concentration of sulfur could not be accurately determined because it was also present in the internal standard used for ICP analysis. Additionally, sulfur was present as a counterion in many of the salts used during medium preparation, resulting in a sulfur concentration that exceeded the bacterial requirements. Therefore, sulfur was not a growth-limiting factor. Furthermore, increasing the concentrations of other medium components containing sulfur as a counterion, as well as using H₂SO₄ for pH control, would further elevate sulfur levels, reinforcing that sulfur is not a growth limiting element.

# Evolution of the CO consumption

During all bioreactor cultivation, CO concentration in the in and outlet gas streams were monitored by mass spectrometry. With that the absolute consumption rate was computed. During cultivation 2, a calibration problem occurred 100 h after the inoculation. This issue was solved at 140 h, but data were not representative during this time.

*
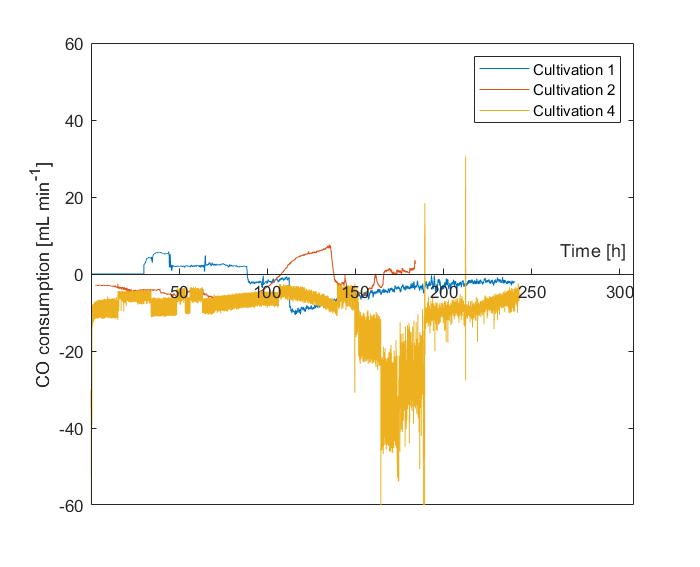
*

Supplementary Figure 2. Time course of the CO consumption of bioreactor cultivations of *H. pseudoflava* with a conventional gas (40% H_2_, 40% CO, 10% CO_2_, 2% O_2_) (Cultivation 1), optimized gas (40% H_2_, 20% CO, 0% CO_2_, 4% O_2_) (Cultivation 2) and conventional liquid medium composition, optimized gas and liquid medium compositions (Cultivation 4)
